# Wilms’ Tumor Gene 1 (WT1) Silencing Inhibits Proliferation of Malignant Peripheral Nerve Sheath Tumor sNF96.2 Cell Line

**DOI:** 10.1371/journal.pone.0114333

**Published:** 2014-12-04

**Authors:** Rosalba Parenti, Venera Cardile, Adriana Carol Eleonora Graziano, Carmela Parenti, Assunta Venuti, Maria Paola Bertuccio, Debora Lo Furno, Gaetano Magro

**Affiliations:** 1 Department of Biomedical and Biotechnological Sciences, Physiology Section, University of Catania, 95125 Catania, Italy; 2 Department of Drug Sciences, Pharmacology and Toxicology Section, University of Catania, 95125 Catania, Italy; 3 Business Unit Oncology, Nerviano Medical Sciences S.r.l., 20014 Nerviano Milano, Italy; 4 Department G.F. Ingrassia, Azienda Ospedaliero-Universitaria “Policlinico-Vittorio Emanuele” Anatomic Pathology, University of Catania, 95125 Catania, Italy; University of Bristol, United Kingdom

## Abstract

Wilms’ tumor gene 1 (WT1) plays complex roles in tumorigenesis, acting as tumor suppressor gene or an oncogene depending on the cellular context. WT1 expression has been variably reported in both benign and malignant peripheral nerve sheath tumors (MPNSTs) by means of immunohistochemistry. The aim of the present study was to characterize its potential pathogenetic role in these relatively uncommon malignant tumors. Firstly, immunohistochemical analyses in MPNST sNF96.2 cell line showed strong WT1 staining in nuclear and perinuclear areas of neoplastic cells. Thus, we investigated the effects of silencing WT1 by RNA interference. Through Western Blot analysis and proliferation assay we found that WT1 knockdown leads to the reduction of cell growth in a time- and dose-dependent manner. siWT1 inhibited proliferation of sNF96.2 cell lines likely by influencing cell cycle progression through a decrease in the protein levels of cyclin D1 and inhibition of Akt phosphorylation compared to the control cells. These results indicate that WT1 knockdown attenuates the biological behavior of MPNST cells by decreasing Akt activity, demonstrating that WT1 is involved in the development and progression of MPNSTs. Thus, WT1 is suggested to serve as a potential therapeutic target for MPNSTs.

## Introduction

Malignant peripheral nerve sheath tumor (MPNST) is an aggressive and rare type of sarcoma, usually arising from peripheral nerves. They can occur sporadically or more frequently (up to 50% of cases) from pre-existing neurofibromas in the context of Neurofibromatosis type 1 (NF1) [1], representing the major cause of mortality in this syndrome [2]–[5]. Nevertheless its pathogenesis is poorly understood. Although genes involved in regulating the cell cycle and growth signal transduction have been reported to be deregulated mainly in MPNST [6]–[8], there is still an urgent need to identify other molecular actors in order to plan new therapeutic approaches.

Wilms’ tumor gene 1 (WT1), which maps to human chromosome 11p13, encodes a zinc-finger transcription factor, firstly identified as a tumor suppressor gene in nephroblastoma or Wilms’ tumor, a pediatric kidney cancer [9]–[10]. A combination of alternative splicing with different post-transcriptional modifications is the basis of the existence of at least 36 isoforms [11]–[13]. This may explain the different and apparently opposing roles in proliferation and apoptosis, depending on cellular context [11]–[18].

In normal tissues, WT1 is an important regulatory molecule involved in cell growth and development [19]–[20]. It is required for normal embryogenesis and influences the correct formation of many organs and tissues, especially urogenital, central nervous systems, heart, spleen, and retina [17]–[18], [21]–[25].

WT1 plays a complex role in tumorigenesis raising the question of whether it is a tumor suppressor gene or an oncogene, or if it has a biphasic function, remains an important and intriguing issue [26]. In fact, it was originally recognized as a tumor suppressor gene because of WT1 mutations were found to cause urogenital diseases and kidney tumors but, in several cases, evidence would suggest an oncogenic role [12]–[13], [20], [27]–[28]. In this regard increased expression of WT1 is associated with the development and progression of different human cancers [20], [29], including carcinomas of the lung [30], breast [31], colon [32], pancreas [33], desmoid tumors [34], hematopoietic system tumors [35]–[36], rhabdomyosarcoma [37]–[39], myofibroblastoma [40], melanoma [41] and brain tumors [42]–[44].

WT1 expression has been also reported in various neuroepithelial tumors including peripheral nerve sheath tumors (neurofibromas and schwannomas) [45]–[47]. By real-time RT-PCR, WT1 overexpression has been described also in MPNST [28] even if correlation with grade of malignancy has not been defined [45].

Recently, functional *in vitro* studies showed that WT1 silencing through an antisense oligomer results in growth inhibition in different cancer cell lines, including breast [48]–[49], lung [50], melanoma [51]–[52], glioblastoma [53]–[54], as well as various types of solid tumors [55] cell lines. Moreover, WT1 silencing reduced *in vivo* the number and growth of visible metastatic tumor foci in the lungs through aerosol delivery of PEI-WT1 RNAi complexes [56].

As the role of WT1 in MPNST is not established, reducing its level by specific RNA interference (RNAi) in established MPNST cell line is helpful for a better understanding of its role in the pathogenesis of these tumors.

In this study, we used a human MPNST cell line (sNF96.2) to investigate whether WT1 silencing by RNAi is capable to suppress the growth of this cell line. Moreover, to understand the molecular mechanisms by which WT1 plays its role, some pathways involved in the regulation of cell cycle were examined. The results show that RNAi directed against the human WT1 gene inhibited effectively the WT1 expression and suppressed the growth of the MPNST cells in both dose- and time-dependent manner. This effect occurs through the down-regulation of PI3K/Akt/cyclin D1 signaling pathway, regulating cell cycle progression, cell proliferation and cell transformation.

## Materials and Methods

### Cell Culture

Human malignant peripheral nerve sheath tumor (MPNST) cell lines, sNF96.2, were obtained from American Type Culture Collection (ATCC). Cell line was cultured in Dulbecco’s modified Eagle’s medium (DMEM), containing 10% heat-inactivated fetal bovine serum (Invitrogen, Carlsbad, CA, USA), 4 mM L-glutamine, 4500 mg/L glucose, 1 mM sodium pyruvate, and 1500 mg/L sodium bicarbonate. Cells were maintained in a humidified 37°C incubator with 5% CO_2_. A sub-cultivation ratio of 1∶3 to 1∶4 twice weekly was performed.

### Immunocytochemistry

For immunocytochemistry experiments, sNF96.2 cells were fixed with 4% formaldehyde for 15 min, permeabilized with 0.1% Triton X-100 for 10 min and washed with PBS. After blocking with 5% normal serum in PBS-Triton X-100 cells were incubated overnight at 4°C with the following primary antibodies: polyclonal anti-WT1 antibody from rabbit (C-19, sc-192, Santa Cruz Biotechnology, Heidelberg, Germany) and monoclonal anti-WT1 antibody from mouse (clone 6F-H2, 05-753, Millipore), used in a dilution of 1∶100 in PBS, 0.1% BSA, 0.1% Triton X-100.

Cells were then washed with PBS for 20 min, followed by a 1-hour incubation with the appropriate fluorescent dye-conjugated secondary antibody. Chromosomal DNA was stained with 4′, 6-diamidino-2-phenylindole (DAPI) and imaging was performed with a Leica fluorescence microscope connected to a digital camera (Spot, Diagnostic Instruments, Sterling Heights, USA) and adjusted for contrast in Corel Draw version 9.

### Subcellular fractionation

sNF96.2 cells were detached by trypsin-EDTA, washed three times with ice-cold PBS, collected and centrifuged in microcentrifuge tubes at 3300 rpm for 10 min at 4°C. The proteins were extracted with a lysis buffer (10 mM Tris-HCl plus 10 mM KCl, 2 mM MgCl_2_, 0.6 mM PMSF and 1% SDS, pH 7.4) enriched with protease and phosphatase inhibitor cocktail tablets (Roche Applied Science). For the subcellular fractionation, aliquots of 10^6^ cells were suspended in 150 µl of buffer A (10 mM Hepes, pH 7.9, 1.5 mM MgCl_2_, 10 mM KCl, 0.5 mM dithiothreitol, 0.2 mM phenylmethylsulfonylfluoride), incubated on ice for 15 min and homogenized by 15 passages through a 25 gauge needle, followed by centrifugation at 12000 rpm for 40 s at 4°C. The supernatants were collected and stored as a cytoplasmic fraction, whereas the pelleted nuclei were washed in 70 µl of buffer A and re-suspended in buffer B (20 mM Hepes, pH 7.9, 25% glycerol, 0.42 M NaCl, 1.5 mM MgCl_2_, 0.2 mM EDTA, 0.5 mM dithiothreitol, 0.5 mM phenylmethylsulfonylfluoride) supplemented with 1X of protease inhibitor cocktail (Roche Applied Science). After 30 min incubation on ice, the nuclear extracts were collected by centrifugation at 12000 rpm for 5 min. The extracts were rapidly frozen and stored at −80°C until processed for Western blot. Before freezing, the protein concentration was estimated using the bicinchoninic acid assay (Pierce).

### siRNA transfection of MPNST cell lines

A total of 5×10^4^ cells were seeded into each well of a 6-well tissue plate. The next day, when cells were 40–50% confluent, the cells were transfected with siRNA against WT1 (siRNA-WT1) (Invitrogen Milan, Italy) as previously reported [41], [48]. siRNA-WT1 consisted of one (s-siWT1) following sequence, 5′-AAAUAUCUCUUAUUGCAGCCUGGGU-3′ (WT1-HSS111388), or a pool (p-siWT1) of the following sequences: (WT1-HSS111388), 5′-UUAAGGUGGCUCCUAAGUUCAUCUG-3′ (WT1-HSS187705), 5′-UUUCACACCUGUAUGUCUCCUUUGG-3′ (WT1-HSS111390), using transfection reagent, LipofectAMINE 2000, at a final concentration of 0.2%. A scrambled stealth RNAi oligonucleotide was used as a control (Invitrogen). All procedures were performed in an RNase-free environment. To minimize the cytotoxicity of the reagent itself, cells were washed with medium without FCS and antibiotics. Cells were harvested at different time points after transfection (48 and 72 hours) with different concentrations of siRNA (25 and 50 nM).

### Western blot analysis

Equal amounts of proteins were boiled in LDS sample buffer (Invitrogen) in presence of 1X sample reducing agent (Invitrogen). Each sample was then subjected to electrophoresis on Bolt 4–12% Bis-Tris Plus Gels (Invitrogen). After electrophoresis, proteins were transferred to a nitrocellulose membrane, in a wet system, and proteins transfer was verified by staining membranes with Ponceau S. Membranes were blocked with Tris buffered saline containing 0.01% Tween-20 (TBST) and 5% non-fat dry milk for 1 hour, and then probed overnight at 4°C with the following primary antibodies: rabbit polyclonal anti-WT1 (C-19:sc-192, Santa Cruz Biotechnology Inc, 1∶200), mouse monoclonal anti-PI3K p110 (D-4:sc-8010, Santa Cruz Biotechnology Inc, 1∶200), rabbit polyclonal anti-AKT (9272, Cell Signaling, 1∶1000), mouse monoclonal anti-pAKT (4058, Cell Signaling, 1∶1000), monoclonal rabbit anti-human Cyclin D1 (M3642, Dako, 1∶500), rabbit polyclonal anti-caspase 3, active (8487, Sigma-Aldrich, 1∶1000), rabbit anti-β-actin (A2066, Sigma-Aldrich, 1∶5000), goat polyclonal anti-Lamin A/C (N-18: sc6215, Santa Cruz Biotechnology Inc, 1∶500). The membranes were rinsed three times in TBST and the appropriate HRP-conjugated secondary antibody (sc-2030, goat anti-rabbit, 1∶20000; sc-2005, goat anti mouse, 1∶5000; sc-2020, donkey anti-goat, 1∶5000, all from Santa Cruz Biotechnology) was incubated for 1 hour at RT. The blots were developed using enhanced chemiluminescent solution (Millipore) and visualized with a chemiluminescent Western blot imaging systems (Alliance, UVITEC). Bands were measured densitometrically, and their relative density was calculated based on the density of the β-actin or Lamin A/C signals in each sample. For extracts from subcellular fractionation, values were expressed as arbitrary densitometric units (A.D.U.) corresponding to signal intensity, while siRNA results were reported as protein fold change vs. scrambled controls.

### Cell proliferation assay

After transfection, siRNA-transfected cells were harvested at specific time points. The total viable cell number was assessed by trypan blue exclusion assay and counted by a hemacytometer under an inverted microscope (Leica).

### Statistical Analysis

In this study, the results are expressed as the means ± standard deviation (SD). All experiments were repeated at least three times. Statistical significance was determined by the two-tailed Student’s t-test, and P-values<0.05 were considered to indicate statistically significant differences.

## Results

### Cell morphology analysis and immunocytochemistry

To investigate the WT1 localization at cellular level, MPNST sNF96.2 cell line was used (ATCC) [57]–[58]. Cells had spindle-shaped morphology and were immunopositive for S-100 indicating Schwann cell lineage.

**Figure 1:**
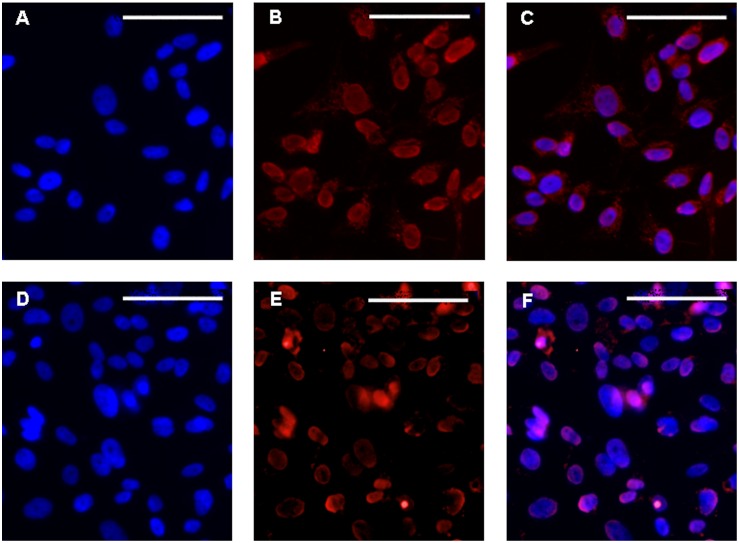
WT1 expression in sNF96.2 cells determined by immunocytochemistry. Immunofluorescence positive cells to 6F-H2 (**B**) and C-19 WT1 (**E**) antibody are merged (**C** and **F**, respectively) with their own DAPI stained nuclei (**A** and **D**). Scale bars: 50 µm.

**Figure 2:**
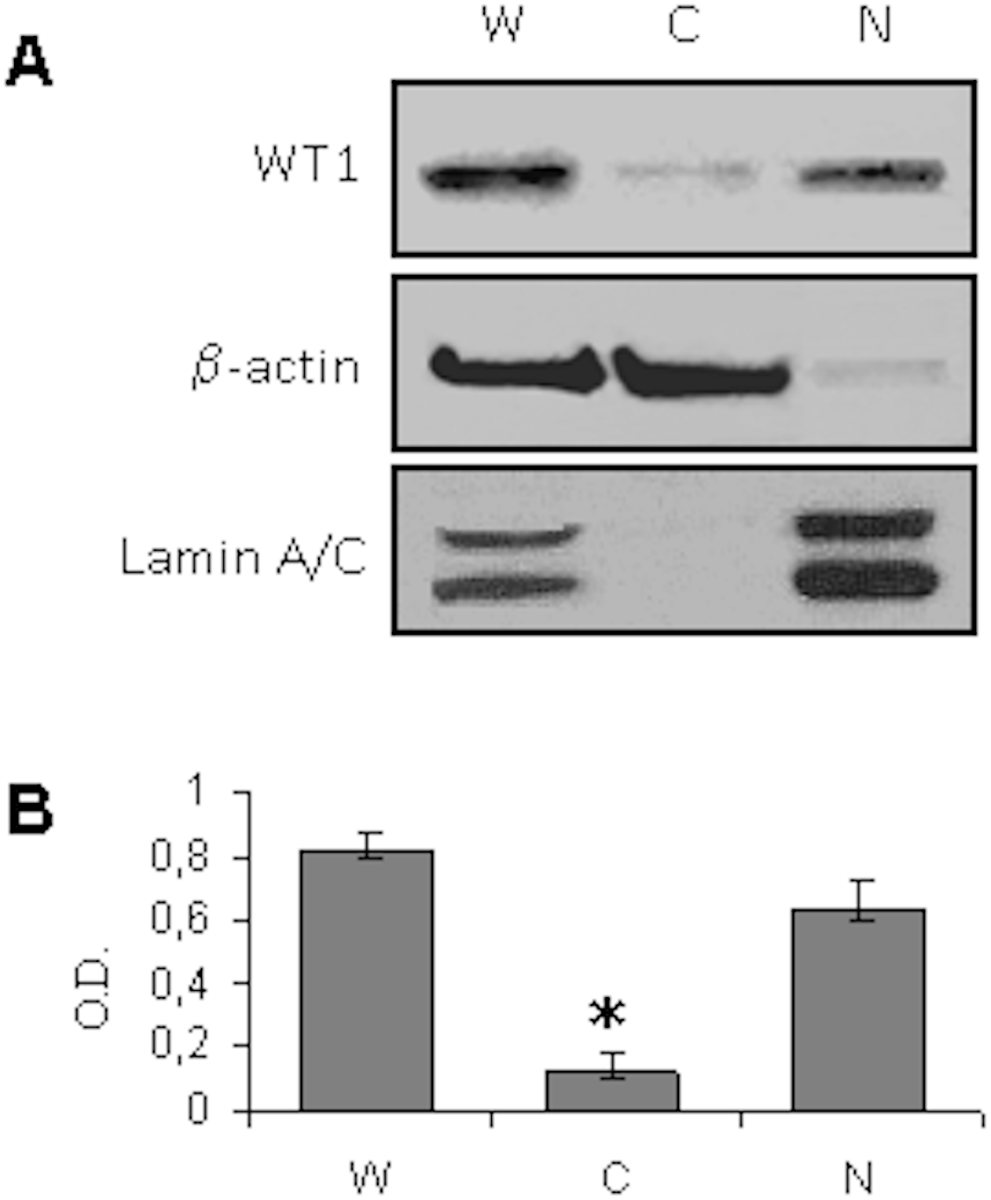
Western blot analysis on cellular proteins separated into nuclear and cytoplasmic fractions from sNF96.2 cells. **A**: Whole cell lysate (W), cytosolic (C), and nuclear (N) fractions were immunoblotted with C-19 WT1 antibody. **B**: Results were expressed as optical density (O.D.). *p<0.05 relative to whole cell lysate.

For immunocytochemistry two different antibodies directed against the C-terminal (C-19, sc-192,) or the N-terminal (clone 6F-H2) portion of the WT1 molecule, respectively, were used. Both antibodies showed similar immunoreactivity even if the specificity of antibody against the N-terminal portion (clone 6F-H2) was higher than WT1 C-19 antibody (Fig. 1). The results revealed that WT1 protein is strongly expressed in the nucleus and in the cytoplasmic area around the nucleus (Fig. 1). To confirm the intracellular distribution of WT1, cellular proteins were separated into nuclear and cytoplasmic fractions using the total cellular lysate as a control. Western blot analysis revealed that WT1 was predominantly located in the nuclear fraction compared to cytoplasmic one, which showed a very weak expression of WT1 (Fig. 2).

### WT1 silencing

**Figure 3:**
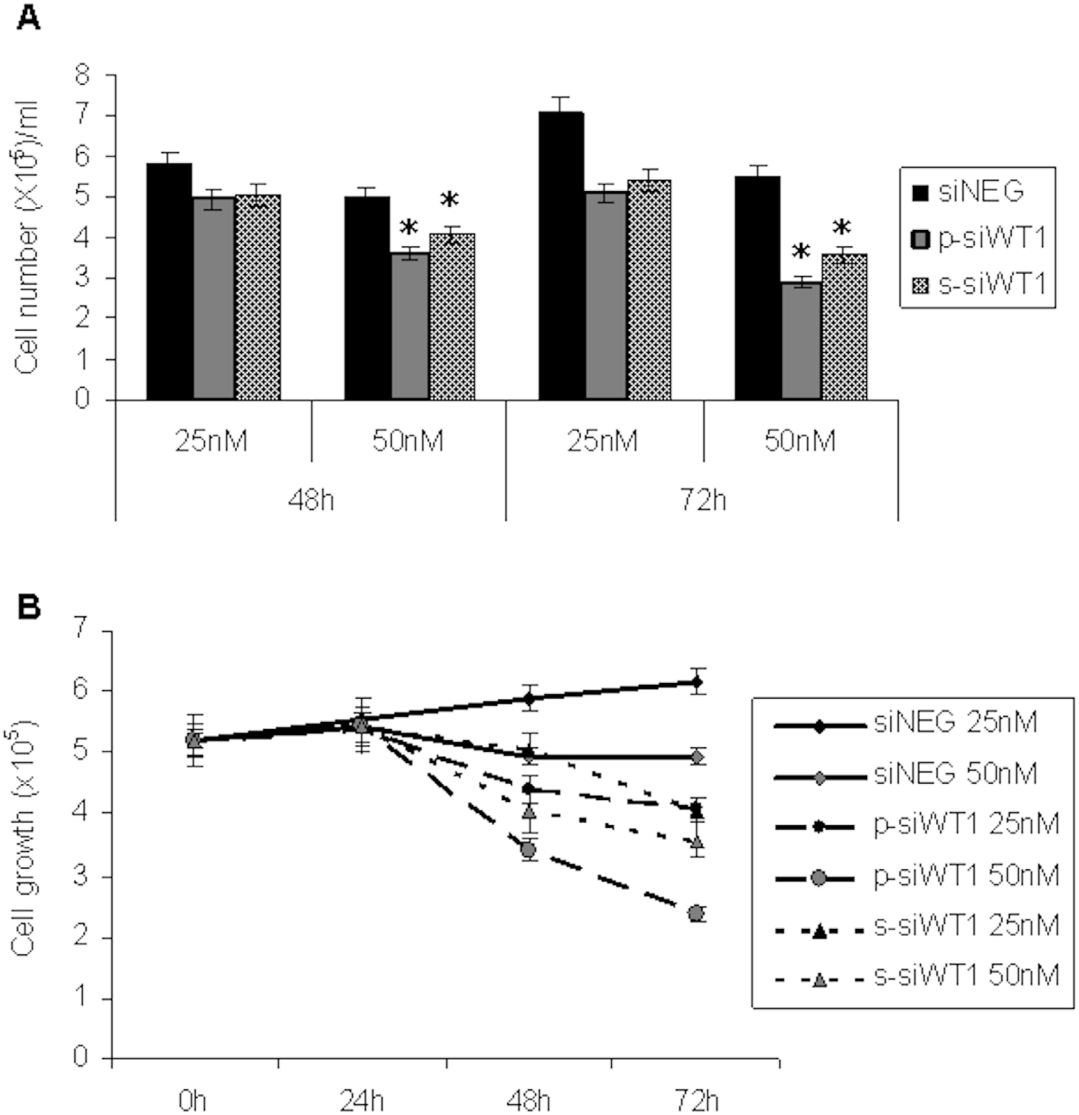
siWT1 on cell proliferation. **A**: Effects of 25 nM and 50 nM p-siWT1 and s-siWT1 on sNF96.2 cell proliferation at 48 and 72 hours. Data represented the average value of three independent transfection experiments. *p<0.05 compared to siNEG ones at same time. **B**: Growth curves of sNF96.2 cells treated with 25 nM and 50 nM siNEG, p-siWT1 or s-siWT1.

**Figure 4:**
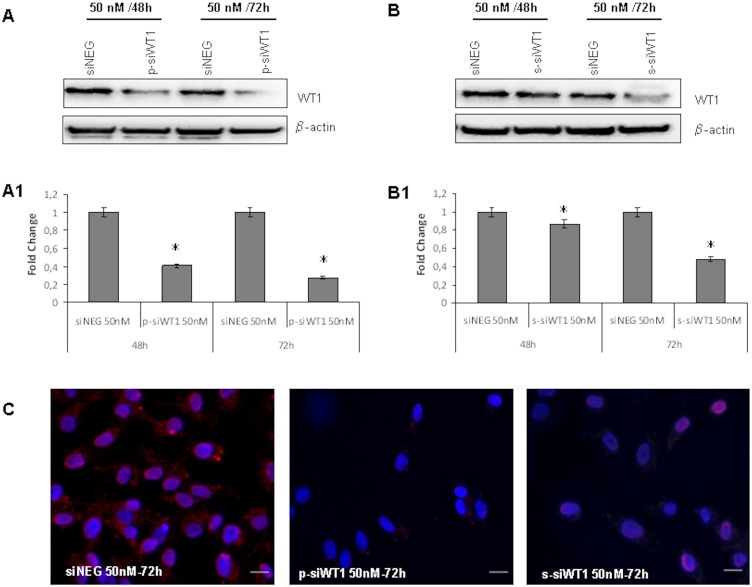
Time-course of siWT1 on sNF96.2. Western blot analysis on cells treated with 50 nM p-siWT1 (**A**) or s-siWT1 (**B**), compared to those treated with scrambled siNEG. siWT1 results were expressed as fold change compared to siNEG ones (**A1** and **B1** for p-siWT1 and s-siWT1, respectively). *p<0.05. **C**: Staining of WT1 protein in sNF96.2 cell treated with 50 nM siNEG, p-siWT1 or s-siWT1. Scale bars: 20 µm.

WT1 silencing (siWT1) experiments were performed to determine their effects on cell viability using scrambled short interfering RNA (siNEG) as a control. sNF96.2 cells were transfected with 25 and 50 nM siWT1, both single (s-siWT1) and pool (p-siWT1), or siNEG for 48 and 72 hours. The results showed that siWT1, inhibited the growth of sNF96.2 cells in a time- and dose-dependent manner decreasing the total number of cells (Fig. 3). In particular, the cell number started to decrease, although at low level, with 25 nM siWT1 (p-siWT1: 15±5%; s-siWT1: 12±5%), and evidently with 50 nM siWT1 (p-siWT1: 28±5%; s-siWT1: 18±5%) after 48 hours treatment (Fig. 3A). Moreover, 72 hours post-transfection, while 25 nM siWT1 still partially inhibited the decrement of cell number (p-siWT1: 29±5%; s-siWT1: 24±5%), 50 nM siWT1 was able to halve significantly the total cell number respect to 50 nM siNEG (p-siWT1: 47±5%; s-siWT1: 35±5%) (Fig. 3A). Thus, to verify the effect of siWT1 (p-siWT1 and s-siWT1) on cell proliferation, growth curves were performed and percentage of cell death calculated (Fig. 3B). The results showed that 50 nM siWT1 inhibited significantly the proliferation of sNF96.2 cells, up to 60% after 72 hours respect to siNEG (Fig. 3B) for both single and pool siWT1 tested. To determine that the inhibition of proliferation was related to WT1 down-regulation, the levels of WT1 protein were monitored by Western blot (Fig. 4A–B) and immunocytochemistry (Fig. 4C). The results showed that WT1 protein expression was clearly inhibited at 50 nM p-siWT1 or s-siWT1 treatment for 72 hours (Fig. 4).

### Effects of siRNA WT1 on apoptosis

**Figure 5:**
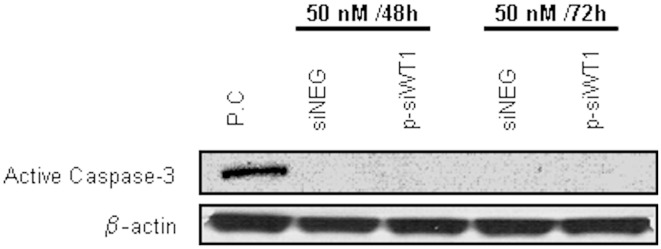
siWT1 on apoptosis. Effects of siWT1 on cleaved caspase-3 expression in sNF96.2 cells treated with 50 nM for 48 and 72 hours determined by Western blot analysis. As a positive apoptosis control (PC), cell lysate of human fibroblasts exposed to 0.1 mM of staurosporine for 24 hours was used [83].

To test the effect of WT1 silencing on apoptosis, we examined the activity of caspases 3, which are effector of the apoptotic pathway. Therefore, we used Western blot analysis to measure the expression of active caspase 3 in sNF96.2 cells treated with siWT1 compared to negative control (Fig. 5). We found that the cleaved caspase-3 expression did not change at 50 nM siWT1 after both 48 and 72 hours of treatment (Fig. 5).

### Effects of siRNA WT1 on cell cycle

**Figure 6:**
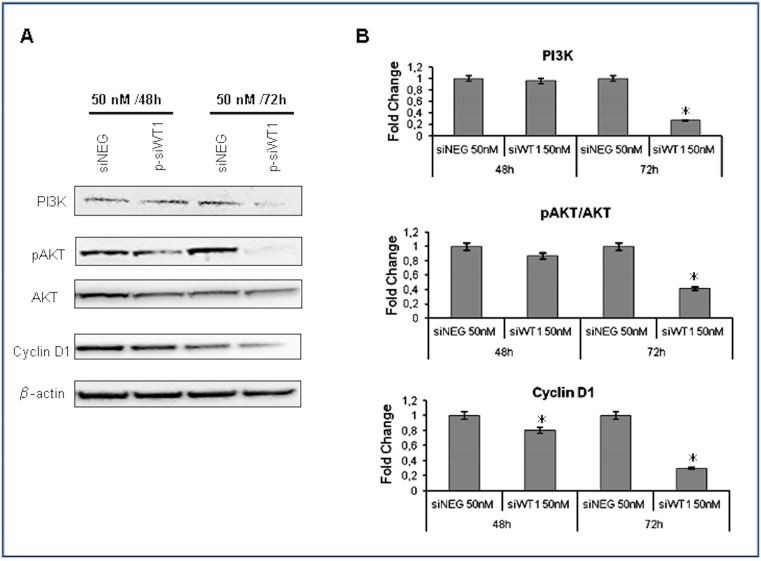
siWT1 on cell cycle. **A**: Effects of siWT1 on PI3K/Akt/Cyclin D1 pathway in sNF96.2 cells treated with 50 nM p-siWT1 for 48 and 72 hours determined by Western blot analysis. **B**: Results were reported as fold change compared to siNEG ones (*p<0.05).

In order to assess the influence of siRNA WT1 on cell cycle, we analyzed the PI3K/Akt/cyclin D1 signaling pathway, which regulates cell cycle progression and is implicated in the cell proliferation and transformation [59]–[60]. Therefore, we used Western blot analysis to measure the expression of PI3K, AKT/pAKT and Cyclin D1 in sNF96.2 cells treated with siWT1 compared to negative control (Fig. 6). At 50 nM siWT1 the expression of PI3K, pAKT and Cyclin D1 proteins slightly decreased after 48 hours to reach a maximum decrement after 72 hours of treatment (Fig. 6).

## Discussion

WT1 involvement in human cancer is very complex, acting as a tumor suppressor in some contexts and as an oncogene in others [61]. Its variable involvement in a large series of tumors is likely due to the complex nuclear/cytoplasmic roles played by WT1 [13], [62]. In fact, besides to the well-known role in transcriptional regulation, WT1 is likely involved in RNA metabolism, translational regulation and association with translating polysomes [62]. The different facets of WT1 are in line with the different nuclear/cytoplasmic expression patterns detected in various tumors by using antibodies directed against the C-terminal portion (WT1 C-19) or the N-terminal portion (WT1 clone 6F-H2) of the molecule [17], [29].

In this study, the expression profile of WT1 in MPNST cell line and the effect of siWT1 on cell growth suggest an articulate work planning performed by WT1 in this specific malignancy.

We showed firstly that WT1 is expressed predominantly in nuclear and perinuclear areas and weaker in the cytoplasm of MPNST cells. These results are in line with WT1 involvement in diverse cellular activities and variable behavior due to the fact that it regulates many genes and it can be modulated by a number of cofactors [13], [63]. Of particular interest is the relationship with actin, described in both nucleus and cytoplasm [64]. Notably, perturbation of the actin cytoskeleton is essential for malignant transformation and WT1 was named as one of the proteins implicated in actin cytoskeletal changes in cancer cells [64]–[65]. WT1 might be a specific adaptor protein that links a specific subset of mRNAs to actin for transporting to the target location and, in turn, actin may act as a cytoplasmic anchor for WT1 [64]. Again, Rong [66] described an intriguing interaction of WT1 with Signal transducers and activators of transcription 3 (STAT3), which is overexpressed or constitutively activated in a variety of human malignancies. Synergistically overexpression of WT1 and STAT3 in tumor development, including Wilms’ tumor, increases the expression level of STAT3 target genes, including cyclin D1 and Bcl-xL, resulting in an advantage of cell proliferation. It is noteworthy that the locations of STAT3 and WT1 protein in primary Wilms’ tumor cells were found mostly located in the nucleus compared to prevalent cytoplasm expression in control normal cells near the tumor [66]. The strong WT1 expression in nuclear compartment of MPNST cell line suggests a similar model in this neoplasm. In support of this data, recently it has been showed that EGFR-STAT3 pathway is necessary for MPNST transformation as demonstrated by the results that STAT3 knockdown by shRNA prevented MPNST formation in vivo, and pSTAT3 fall in vivo by reduced EGFR activity [67]. Finally, the concentration of WT1 in the perinuclear zone of MPNST cell lines is consistent with its function close to the nucleus to act in nuclear-cytoplasmic shuttling under appropriate conditions [62]. It is evident that the field of action of WT1 is very broad. Further studies are required to identify the interaction partners of WT1 and to explore the functional relevance of such cooperation for planning new experimental approaches.

In this study we further investigated the significance of WT1 expression in MPNST cell line by WT1 silencing experiments. WT1 knockdown performed in different cancer lines showed to impede cell proliferation and viability by a multitude of mechanisms. Firstly, WT1 downregulation induced mitochondrial damage and resultant apoptosis in different solid tumors [50]–[52], [55]. Numerous studies have demonstrated that WT1 regulates apoptosis by targeting directly or indirectly bcl-2 family members, including the pro-apoptotic family members Bak and Bax, and the anti-apoptotic family member Bfl-1/A1 critically depending on cell lineage analyzed and specific WT1 isoform acting [68]. Differently, WT1 silencing in glioblastoma causes decreased viability by IFG-1R overexpression, which causes a non-apoptotic, non-autophagic programmed cell death termed “paraptosis” [54]. Silencing of WT1 causes decreased proliferation and viability in most cancer cell lines including K562 and MM6 leukemia [69], MCF-7 breast cancer [48]–[49], A549 lung cancer [50], B16F10 melanoma lung metastasis [51], and U251MG human multiform glioblastoma [53]. Accordingly, in MPNST cell line we showed that silencing of WT1 effectively inhibited WT1 protein expression. While activation of apoptosis was not shown, we found a reduced proliferative capacity likely due to WT1 effect on cell cycle progression through a decrease in the protein levels of the key components of PI3K/Akt/Cyclin D1 pathway. The result is in line with previous data showing WT1 effects on the cell cycle both directly and indirectly by regulating genes involved in cell cycle regulation [70]–[73]. PI3K/Akt/Cyclin D1 pathway is now recognized as one of the most important pathways in regulating cell survival and proliferation [74]. Activation of PI3K, an intracellular signal transducer enzyme, can phosphorylate phosphatidylinositol 4,5-biphosphate (PIP2) into phosphatidylinositol 3,4,5-triphosphate (PIP3). As a second messenger, PIP3 recruits Akt to the cell membrane where Akt is fully activated by phosphorylation at position Ser473 [75]. After activation, Akt translocates to the cytoplasm and nucleus to phosphorylate its substrates and promote cell proliferative and survival signals through the upregulation of cyclinD1 [76]–[79]. In conclusion, our results indicate that WT1 knockdown attenuates the biological behavior of MPNST cells by decreasing Akt activity, demonstrating that WT1 is involved in the development and progression of MPNSTs. In models of neuronal differentiation, it has been proposed that WT1 could maintain cells in an undifferentiated state [22], [53], [80]. In this way, silencing of WT1 has been suggested to promote a more differentiated phenotype of astrocytoma cells with a lower proliferative capacity [53]. Likewise, maintaining Schwann cells in more a undifferentiated state might be caused by WT1 in vivo overexpression in human MPNSTs as suggested by the in vitro growth inhibition and reduced cyclin D1 protein levels, caused by WT1 silencing and the expression profile during peripheral nervous system development where the tumor is found.

In conclusion, the present study showed that in MPNST, at least in vitro, WT1 acts as an oncogene rather than a tumor suppressor. It is noteworthy that, AKT and PI3K pathways were recently found to be highly activated in MPNST cell lines so that AKT activation blockade, either by inhibition of the PI3K upstream or directly through AKT inhibitors, may potentially be pursued as a systemic anti-MPNST approach [81]–[82]. Our results suggest that, WT1, intimately influencing pAKT/Cyclin D1 pathway, also could be tested as potential agent for a gene-targeted therapy approach for the treatment of MPNSTs, which represent a human inauspicious disease without still effective targeted therapies.
